# NLC-Based Sunscreen Formulations with Optimized Proportion of Encapsulated and Free Filters Exhibit Enhanced UVA and UVB Photoprotection

**DOI:** 10.3390/pharmaceutics16030427

**Published:** 2024-03-20

**Authors:** Margarete M. de Araújo, Andressa C. Schneid, Mariana S. Oliveira, Samuel V. Mussi, Miller N. de Freitas, Flávia C. Carvalho, Edson A. Bernes Junior, Renato Faro, Hatylas Azevedo

**Affiliations:** 1 Aché Laboratórios Farmacêuticos, Rod. Pres. Dutra-Porto da Igreja, Guarulhos 07034-904, SP, Brazil; andressacschneid@gmail.com (A.C.S.);; 2 Ferring Pharmaceuticals, Av. Engenheiro Luis Carlos Berrini—Cidade Monções, São Paulo 04571-010, SP, Brazil

**Keywords:** nanostructured lipid carrier, skin penetration, in vitro photoprotection, in vivo photoprotection, sunscreen, industrial research

## Abstract

The topical use of sunscreens is recommended for avoiding the damaging effects of UV radiation. However, improvements are still needed in the existing products to enhance their photoprotection effectiveness and safety. This involves minimizing the use of chemical UV filters while providing enhanced and prolonged photoprotection. This work investigated novel sunscreen formulations and their UV protection effects by encapsulating Uvinul^®^ A, Tinosorb^®^ S, and Uvinul^®^ T150 into nanostructured lipid carriers (NLCs) based on bacuri butter and raspberry seed oil. First, the impact of critical formulation and process parameters on NLCs’ particle size was evaluated using a 2^2^ Face Centered Central Composite Design. Then, formulations were evaluated in terms of critical quality factors, in vitro skin permeation, and in vitro and in vivo photoprotection activities. The developed NLCs-containing formulations exhibited appropriate size (122–135 nm), PdI (<0.3), encapsulation efficiency (>90%), and drug content (>80%), which were preserved for at least 90 days under different stability conditions. Moreover, these NLCs-based formulations had equivalent skin permeation to emulsion-based controls, and the addition of NLCs into sunscreen cream bases in the optimum proportion of 20% (*w*/*w*) resulted in enhanced UVA and UVB photoprotection levels, despite a 10% reduction in the total filters content. Altogether, these results describe the application of nanoencapsulated organic UV filters in innovative sunscreen formulations to achieve superior photoprotection and cosmeceutical properties.

## 1. Introduction

**Figure 1:**
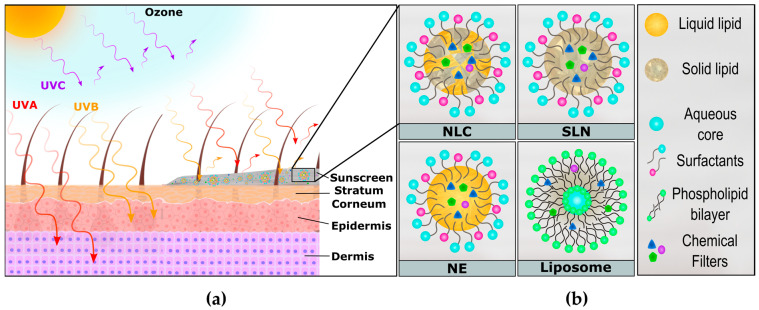
(**a**) Scheme describing the interaction between UVR (UVA, UVB, and UVC) and the different layers of the skin structure (stratum corneum, epidermis, and dermis). (**b**) Depiction of different lipid-based nanoparticles applied to sunscreen formulations.

Life on Earth is a direct product of the sunlight’s extensive impact on biological, chemical, and physical processes. Human health highly benefits from sunlight exposure since vitamin D synthesis is dependent on ultraviolet radiation (UVR) [1]. However, the skin can also be significantly harmed due to sunlight overexposure [2]. UVR is divided into three radiation ranges known as UVA (320 to 400 nm), UVB (290 to 320 nm), and UVC (200 to 280 nm). While UVC is blocked by the ozone layer, UVA and UVB reach different skin fractions, promoting distinct skin damage (Figure 1a). UVA can access the basal epidermal layer and even dermal fibroblasts and is one of the main causes of skin aging and pigmentation. This radiation can produce free radical oxygen species (ROS) and induce DNA damage, ultimately leading to skin cancer [1,3,4]. In parallel, UVB possesses a restricted penetration into the skin, reaching only the epidermal layers, where it can cause skin sunburns and DNA strand breaks [1,5]. Last, UVC radiation stands out as the most energetic among UV radiations, but fortunately, the ozone layer effectively blocks it, making it a relatively lower health concern [1,5,6]. Photoprotection is of utmost importance for skin damage prevention, and the mechanisms underlying this effect are rooted in two main factors. Primary factors are based on sunscreens that contain physical (UVR reflection and scattering) and chemical barriers (UVR absorption) [7,8]. Secondary factors include the action of antioxidants, osmolytes, and DNA repair enzymes, which help to mitigate skin damage by disturbing the photochemical cascade that occurs due to UV sunlight [7,8]. 

There has been extensive research into sunscreen formulations over the past 30 years, involving the exploration of various primary protective factors, such as organic and inorganic components, to enhance sun protection factors (SPF). Organic UV filters, also known as chemical UV filters, can absorb UV and/or visible radiation, hence, they cover a broad region of the spectrum when combined, decreasing the radiation dose able to harm the skin [6,9]. Nonetheless, several organic UV filters and their degradation products may cause allergic dermatitis, and due to their small size and interaction with skin components, they might penetrate the stratum corneum and diffuse through the subsequent skin layers, which could lead to undesired systemic absorption [5,10]. A second approach is the use of physical filters like ZnO, TiO_2_, and/or iron oxide particles that can absorb, scatter, and reflect UVR [6]. Initially, products using inorganic UV filters were based on microparticles, which promoted intense light scattering and reflection, resulting in an opaque white appearance on the skin [1,6]. To solve this, the next generation of sunscreens containing ZnO and/or TiO_2_ were developed as nanoparticles to decrease this opacity effect [1,11]. Overall, the safety of inorganic UV filters in the microparticle size range is acceptable, given their low percutaneous absorption [1,11,12]; however, inorganic nanoparticles can accumulate in the follicular region and might migrate to deeper layers of the skin [13,14,15,16,17]. Inorganic nanoparticles are particularly not recommended to be employed in spray formulations, because their inhalation can lead to lung inflammation and microvascular dysfunction [1,15]. To overcome these hurdles, researchers have been investigating lipid-based nanoparticles as safer and biodegradable alternatives for encapsulating organic UV filters to benefit from the combined effects promoted by free organic UV filters and nanostructures [18,19,20,21]. For instance, a 2-fold increase in SPF was reported when 20% (*w*/*w*) of a lipid-based nanocarrier was added to a sunscreen formulation [18].

The use of lipid nanoparticles (LNs) (Figure 1b), such as solid lipid nanoparticles (SLNs), nanostructured lipid nanocarriers (NLCs), nanoemulsions (NEs), and liposomes, has been used as a successful strategy for SPF enhancement [1,9,19]. Their small particle size ensures close contact with the stratum corneum, leading to an occlusive effect on the skin surface due to lipid film formation and reduced transepidermal water loss (hydrating effect) [1,9,20]. Among the available LN platforms, NLCs seem to exhibit enhanced (photo)stability compared to SLNs and NEs [21,22] and better scalability in comparison to liposomes [19]. The hurdle faced by SLNs is the unexpected leakage of encapsulated species, which is the result of the solid lipid crystallization that drives these molecules to the outer fraction of the nanostructure, decreasing its stability [19]. In parallel, NEs can be subjected to higher lipid peroxidation under UVA exposure than NLCs [22]. The employment of NLCs can increase filters’ chemical stability by precluding the direct access of radiation to them [23,24]. Additionally, it may enable an extended photoprotection effect due to several factors, including (i) sustained release: in contact with the skin lipids, the NLCs’ structure can lixiviate, delivering the organic UV filters in a controlled and sustained way [25,26]; (ii) residence time: the lipophilic nature of NLCs can enhance their interaction with the skin surface compared to free filters (organic and inorganic) and consequently increase their residence time [25]; (iii) low skin penetration/diffusion: in addition to their strong interaction with lipophilic skin components, NLCs typically have diameters exceeding 40 nm, impeding their diffusion through the aqueous nanopores dispersed in multiple layers of the skin [1,4,25].

In this study, we developed innovative sunscreen formulations using NLCs (SC-NLC) based on bacuri butter and raspberry seed oil. To achieve industry standards, the SC-NLC’s production was optimized by investigating the composition and process parameters by QbD. The stability and skin penetration of SC-NLC were assessed to evaluate its integrity and safety, and the photoprotective effects of the nanoformulation were confirmed using in vitro and in vivo assays. Furthermore, we describe several steps commonly required to enable the translation of the product to an industrial setting, following the main international guidelines for sunscreens and nanotechnology from the FDA—U.S. Food and Drug Administration, COLIPA—The European Cosmetic and Perfumery Association, and SCCS—Scientific Committee on Consumer Safety [27,28,29]. We aimed to shed light on the path toward translating nanomaterial-based sunscreen formulations from bench to counter [30,31].

## 2. Materials and Methods

### 2.1. Materials

Bacuri butter (*Platonia insignis*) was supplied by Beraca (São Paulo, Brazil—SisGen Register Number: AB261F8) and raspberry seed oil (*Rubus idaeus* L.) by Distriol (São Paulo, Brazil). Polyglyceryl-3-dioleate (Plurol^®^ oleique) was gently donated by Gattefossé (Saint-Priest, France), and Polysorbate 60 (Tween^®^ 60) by Merck (Darmstadt, Germany). Additionally, Uvinul^®^ A Plus (diethylamino hydroxybenzoyl hexyl benzoate), Tinosorb^®^ S (bis-ethylhexyloxyphenol methoxyphenyl triazine), and Uvinul^®^ T150 (ethylhexyl triazone) were kindly donated by BASF (Mannheim, Germany). HPLC-grade ethanol and dimethylformamide (DMF) was procured from Merck (Darmstadt, Germany), acetonitrile (ACN) from J.T. Baker (Phillipsburg, NJ, USA), and phosphoric acid from Sigma-Aldrich (St. Louis, MO, USA). The purified water was obtained from a MilliQ^®^ system from Millipore (Burlington, MA, USA). 

### 2.2. Emulsion Preparation

The NLCs’ dispersion was composed of a mixture of bacuri butter and raspberry seed oil summing 10% of lipid content, Polyglyceryl-3-dioleate, Polysorbate 60, and, with and without the filters, Uvinul^®^ A Plus, Tinosorb^®^ S, and Uvinul^®^ T150 in a ratio of 3:2:1, respectively. Firstly, all the components were mixed in a glass container and heated in a water bath at a temperature above the melting point of the solid lipid. After the mixture was completely melted, water was added at temperatures ranging from 70 to 80 °C, followed by 2 min of stirring using an Ultra-Turrax™ blender at 12,000 rpm.

### 2.3. NLCs Preparation Using Ultrasonication Method

The NLCs were prepared immediately after the obtention of the emulsion and then submitted to high-intensity probe sonication (20% amplitude) for 10 min, using a high-intensity ultrasonic processor (CPX 500 model, Cole-Palmer Instruments, Vernon Hills, IL, USA).

#### 2.3.1. Factorial Design for NLC Formulation

For this study, a 2^2^ Face Centered Central Composite Design (CCF) was carried out with five replicates at the central point. The variables (x_1_ and x_2_) were the percentage of Polysorbate 60 and Polyglyceryl-3-dioleate in the formulation, respectively. The total lipid load (10%—the proportion of bacuri butter and raspberry seed oil is described in patent application WO2023137532A1) and the total of filters (6%) were considered as main constants; also, the total amount of surfactant was not included as a variable in this study due to its high correlation (Pearson test, r = 0.88) with the Polysorbate 60 percentage (x_1_). The excipient identity and their range of use were defined in previous studies carried out in our research group. The significant variables to be applied in the polynomial fit and the model’s lack of fit were indicated by the analysis of variance (ANOVA, 95% confidence) using Design Expert 13.0 Software (Minneapolis, MN, USA). The values of each factor level and their combination are shown in Table 1 and Table 2, respectively. The evaluated response or Critical Quality Attribute (CQA) was the NLCs’ diameter, and its desired target was values ranging from 100 to 300 nm.

#### 2.3.2. NLCs Preparation Using a High-Pressure Homogenizer

The NLCs were also produced using a high-pressure homogenizer (GEA Panda Plus 2000, Parma, Italy) to provide an optimized formulation obtained through a process that can be easily scaled up in pharmaceutical industries. These samples were also obtained using the emulsion described in Section 2.2. The process optimization is described below.

### 2.4. Factorial Design for NLCs Production Process

The optimization of the NLCs production process was carried out through a 2^2^ CCF, with triplicates at the central point, where the variables were pressure applied (x_1_) and time the formulation was submitted to homogenization (x_2_). This study used an optimized sample with characteristics within the desired region. As aforementioned, the significant variables and the model’s lack of fit were determined by ANOVA (95% confidence), using Design Expert 13.0 Software (Minneapolis, MN, USA), using a lambda value of −1.67. Table 1 shows the values of the factors’ levels. Finally, the response (CQA) evaluated was also the particle diameter, targeting sizes between 100 and 300 nm.

### 2.5. Determination of Particle Size, Polydispersity Index, Zeta Potential, and pH

The dynamic light scattering (DLS) and zeta potential (ZP) measurements of the particles were carried out in a Malvern Zetasizer Ultra (Worcestershire, United Kingdom). The DLS was performed in backscattering mode (detection angle = 173°). The correlation curves were analyzed by applying cumulant analysis to obtain Z-average hydrodynamic diameters; to define size distributions, a non-negative least square fitting algorithm (general purpose) was applied, which was implemented by Malvern Software (version 3.10). The samples were analyzed in a dilution of 200 times; the diluents used were water (DLS) and a solution of KCl 10 mmol/L (ZP). The pH values of the SC-NLC formulation were assessed by a pH meter (Mettler Toledo, Columbus, OH, USA) at room temperature. All the measurements were performed using three replicates.

### 2.6. Ultraviolet-Visible Spectroscopy

The UV-Vis absorption of formulation components with and without Uvinul^®^ A Plus, Tinosorb^®^ S, and Uvinul^®^ T150 was performed in a spectrophotometer (Mettler Toledo, Columbus, OH, USA) model UV-Vis IV5, in the spectrum range from 280 nm to 400 nm. The samples were prepared by solubilizing the filters with and without NLCs and bare-NLCs in THF.

### 2.7. Atomic Force Microscopy (AFM)

The morphology of the optimized SC-NLC was evaluated by AFM. The sample was prepared by depositing dilute NLCs dispersions on a freshly cleaved mica plate and drying with argon. Images were acquired using a Shimadzu Scanning Probe Microscope SPM-9600 model (Kyoto, Japan) equipped with a 100 μm tripod scanner and pyramidal cantilevers with silicon probes (force constant: 10–130 N/m) at a resonance frequency of 204–497 kHz. The measurements were carried out in intermittent contact mode at a scan speed of approximately 1 Hz to avoid damage to the sample surface.

### 2.8. Rheology

To find the fluid flow and viscosity curves of the SC-NLC formulation, mixtures of 80% cream bases (described in Section 2.12) and 20% SC-NLC or 20% purified water were prepared. The data acquisition to determine the cream bases mixture flow and viscosity behavior was performed in an Anton Paar MCR302 Rheometer (Graz, Austria), using aluminum parallel plates geometry of 25 mm of diameter (PP25/S) and a gap of 1 mm. All measurements were performed at 25 °C. Continuous flow measurements were carried out by increasing the shear rate from 0.01 to 200 1/s and, hence, determining the viscosity. SC-NLC formulation flow and viscosity curves were obtained using an Anton Paar ViscoQC 300 viscometer (Graz, Austria), using a spindle DG26 with a DIN adapter. The measurements were performed at 25 °C. Viscosity and shear stress were measured with a rising shear rate from 12.91 to 129.1 1/s.

### 2.9. Analytical Method for Quantification of Uvinul^®^ A Plus, Uvinul^®^ T150, and Tinosorb^®^ S in NLCs

The method presented in this study was based on national and international guidelines for the quantification of Uvinul^®^ A Plus, Uvinul^®^ T150, and Tinosorb^®^ S in NLCs formulations [32].

#### 2.9.1. Standard and Sample Preparation

The standard solution was a mixture of Uvinul^®^ A Plus, Tinosorb^®^ S, and Uvinul^®^ T150 with concentrations of 48, 32, and 16 µg/mL, respectively, dissolved in a diluent solution of ethanol/DMF (1:1, *v*/*v*). Then, 1 mL of this solution was added to a 5 mL volumetric that had its volume completed using the diluent solution. Afterward, 400 µL of the latter solution was added to a 5 mL volumetric flask, which was filled up using the diluent solution. This solution was stored in an amber flask, protected from light, and used within three days. The sample preparation was performed as follows: (1) 100 µL of NLCs dispersion containing the chemical filters was added to a 5 mL volumetric flask, followed by 2 mL of acetonitrile. After manual stirring, the flask was filled up to 5 mL with the diluent solution; (2) 400 µL of solution 1 was added to a 5 mL volumetric flask, which had its volume completed by the diluent solution. The theoretical concentration of Uvinul^®^ A Plus, Tinosorb^®^ S, and Uvinul^®^ T150 in the sample solution is 48, 32, and 16 µg/mL, respectively.

#### 2.9.2. Equipment and Analytical Method

The experiments were performed in an Agilent 1260 liquid chromatographic system (Santa Clara, CA, USA), with a quaternary pump 1260 Flexible Pump (Model G7104C) coupled with a diode array detector 1290 DAD FS (Model G7117A). Openlab CDS EzChrom V A 04.08 Software was used to acquire and process the data. The column Waters XBridge BEH C18 (150 mm × 4.6 mm, 3.5 µm, Waters, Milford, MA, USA) was used to separate the chemical filters. The column was thermostated at 35 °C (±1 °C) using a 1260 MCT oven (Model G7116A) throughout the analyses. This method used a gradient mixture of ethanol—mobile phase A, and acidified water with pH 3.5, obtained using phosphoric acid 1%—mobile phase B. The mobile phase flow was 0.7 mL/min, and the chromatographic run window was 31 min. The injection volume and wavelength where the chemical filters were detected were 20 µL and 312 nm, respectively [32]. The solutions prepared for HPLC analysis were filtered using a 0.45 µm PVDF (Hydrophilic Polyvinylidene Fluoride) filter. This analytical method was developed and validated. 

#### 2.9.3. Encapsulation Efficiency and Loading Capacity of the Chemical Filters in the NLCs

The encapsulation efficiency (%EE) of Uvinul^®^ A plus, Uvinul^®^ T150, and Tinosorb^®^ S into the NLCs was carried out by the ultrafiltration–centrifugation method, where regenerated cellulose filters with molecular exclusion pore size of 100 kDa (Millipore, (Burlington, MA, USA)) were used. The centrifugation (660 g for 10 min) was carried out using a mix of 1 mL of SC-NLC and 1 mL of diluent, followed by the collection of 16 µL of the liquid in the bottom compartment of the tube. The concentration of each chemical filter was analyzed by HPLC, following the method described in Section 2.9.2. The encapsulation efficiency (%EE) and loading capacity (%LC) of the SC-NLCs for Uvinul^®^ A Plus, Uvinul^®^ T150, and Tinosorb^®^ S were determined according to Equations (1) and (2), respectively:

%EE = [(F_total_ − F_free_)/F_total_] × 100% (1)

%LC = [(F_total_ − F_free_)/L_total_] × 100% (2)

where F_total_ is the total concentration of the given chemical filter in the formulation, F_free_ is the concentration of the non-encapsulated chemical filter collected in the ultrafiltration-centrifugation process, and L_total_ is the total concentration of lipids in the formulation [33].

### 2.10. Physicochemical Stability Study

The physicochemical stability of the final NLCs formulation was monitored for three months at temperatures of 4, 25, and 40 °C and relative humidity of approximately 75%. The parameters followed were particle size, PdI, zeta potential, pH, total content, and encapsulation efficiency (%EE). All the measurements were performed using three replicates. Analysis of variance (ANOVA, 95% confidence level) was used to compare the significant differences over time. The method employed in this study was based on the Brazilian Health Regulatory Agency (ANVISA) and ICH (International Council for Harmonization of Technical Requirements for Pharmaceuticals for Human Use) guidelines [34,35]. 

### 2.11. In Vitro Skin Permeation Study 

In vitro permeation studies were conducted following the protocol outlined in OECD Guideline 428 [36]. Explants of human skin obtained in optional abdominoplasty surgeries of healthy patients (male or female) were used as the biological membrane in this study. After the surgery, the skin was stored in NaCl 0.9% solution and kept under refrigeration for up to 24 h. The use of these explants of human skin was approved by the Research Ethics Committee of the São Francisco University (São Paulo/SP—Brazil), number 5.503.565, and the Certificate of Ethical Appreciation, number 56005722.8.0000.5514. The experiment was carried out in a DHC-6T Dry Heat Transdermal System (Logan Instruments Corp., Somerset, NJ, USA) in quadruplicate. In the ex vivo experiments, the skin explant (1.76 cm^2^ of diffusion area) was placed in between the Franz cell donator fraction and the receptor fraction, where the internal portion of the hypodermis was exposed to a sodium phosphate buffer solution, inserted into the system without air bubbles. Afterward, the surface of the skin explant was covered with 300 µL of sample (SC-NLCs or emulsion) in the Franz cell device. The experiment was performed for 12 h at 37 °C [36]. Next, the dermis and epidermis of each replicate were mechanically separated by heating the skin sample in a water bath at 50–60 °C. Afterward, the UV filters were extracted using 5 mL of a solution of ethyl acetate/methanol (1:1, *v*/*v*) and submitted to 2 min of stirring in a vortex and 40 min in an ultrasonic bath, followed by 10 min of centrifugation at 1500 rpm. Following this, the supernatant of the sample was further analyzed by HPLC. The receptor liquid was also sampled for further analysis. Then, the concentration of Uvinul^®^ A Plus, Tinosorb^®^ S, and Uvinul^®^ T150 contained in each skin explant and in the receptor liquid were determined by HPLC (Agilent 1100 HPLC System, DAD G1315B), using the column RP-C18 Luna Phenomenex, in isocratic mode, mobile phase flow of 0.8 mL/min, and detection wavelength of 250 nm. The LOQ of this method was 0.35291, 0.00084, and 0.00033 µg/mL for Uvinul^®^ A Plus, Tinosorb^®^ S, and Uvinul^®^ T150, respectively.

### 2.12. Photoprotection Efficacy Studies

The in vitro and in vivo photoprotection properties were evaluated for the optimized NLCs formulations and the respective control emulsions when added to selected pigmented and non-pigmented cream bases in the proportions of 10, 20, and 30% for in vitro tests and 20% for in vivo assays. The assembled tests carried out in this study followed the requirements mentioned in the international guidelines (FDA and SCCS) [27,29], ISO (International Organization for Standardization) 24442:2022 (UVA-PF), and 24444:2019 (SPF) [37,38].

#### 2.12.1. In Vitro Photoprotection Test

The in vitro test of photoprotection efficiency against UVA and UVB radiation was carried out by diffuse transmittance using an integrating sphere (UV-2000S Ultraviolet Transmittance Analyzer, Labsphere, North Sutton, NH, USA) to properly assess the light absorption and scattering after interacting with the samples. Polymethymetacrylate (PMMA) plates were used as substrates, and the sample application ratio was 1.3 mg/cm^3^. The analyses were performed in triplicate, and nine points per plate were measured in each sample, from 290 to 400 nm. The substrate without a sample was used as reference support.

The samples that were analyzed were (I) SC-NLC 100%, (II) Emulsion 100% (obtained before the high-pressure homogenization process—presenting size of several micrometers), (III) Cream base SPF 30 and UVAPF 10 (CB-I), (IV) Cream base 80% (diluted with water), (V) SC-NLC 20% (*w*/*w*) in the cream base, (VI) Emulsion in a cream base in the proportion of 20% (*w*/*w*). The cream base (CB-I) presented in its composition 13.5% of chemical filters (ethylhexyl methoxycinnamate, bis-ethylhexyloxyphenol methoxyphenyl triazine—Tinosorb^®^ S, diethylamino hydroxybenzoyl hexyl benzoate—Uvinul^®^ A Plus, ethylhexyl triazone—Uvinul^®^ T150).

#### 2.12.2. In Vivo Photoprotection Test

The in vivo photoprotection test was performed in humans, with the study population being ten females aged between 18 and 70 years old with healthy skin. The volunteers presented skin phototype (Fitzpatrick classification) (SPT) class II-III, and III-IV, for SPF and UVA-PF evaluation, respectively. The ratio of volunteers of SPT II and III for the SPF testing was 1:4 (II:III), whereas for the UVA-PF assay, the SPT III:IV ratio was 4:6. First, in a pre-test to determine the minimal erythemal dose (MED), each participant had six sites (35 cm^2^, 1 cm a part) of their back skin exposed to UV radiation from 2 to 4 h. Then, six other sites were delimited (35 cm^2^, 1 cm apart) for the samples’ application. In this step, the previously homogenized samples were spread onto each site in the proportion of 2 ± 0.05 mg/cm^2^ or a total weight of 70 mg (±2.5%) under dark light. The samples were let dry for 15 to 30 min at temperatures between 16 and 25 °C. The values for MED of protected skin were obtained by applying doses of the samples in six sub-sites in a geometric progression of 1.25; consequently, the variation dose between sites was 25%. The median dose was defined based on the MED obtained for unprotected skin. The skin sites used for the pre-test and test were localized next to the participant’s spine and in-plane regions, which were analyzed horizontally with the participant lying down. The instruments used to perform the sun simulation were the Solar Simulator multi-port 601-300W (Solar Light CO^®^ Glenside, PA, USA), UVA radiometer model PMA 2113 or 2118 (Solar Light CO^®^), and DCS model PMA 2100 version 1.16 (Solar Light CO^®^). The solar simulator promoted continuous light emission in the UVB and UVA spectrum, varying from 290 to 400 nm. The values of UVA-PF were calculated as follows:

UVA-PF_i_ = MED_protected_/MED_unprotected_ (3)

UVA-PF_mean_ = ∑PFUVA_i_/n (4)

where UVA-PF_i_ is the individual photoprotection against UVA radiation, MED_protected_ and MED_unprotected_ are the minimal erythemal doses in protected and unprotected skin, respectively. The UVA-PF_mean_ is the mean photoprotection against UVA radiation based on the number (n) of participants in the study [37,38]. The methods to determine the SPF in vivo request a minimum of ten valid results, which is true when the 95% confidence interval of the SPF mean is within 17% [37,38]. Thus, we certify that this condition was followed to guarantee data integrity.

Several sample groups were included in this assay; they were (a) 80% cream base + 20% purified water, (b) 80% cream base + 20% emulsion, and (c) 80% cream base + 20% SC-NLC. Three cream bases were used in this study, and they were labeled CBI (described in Section 2.12.1), CBII (FPS 30 and UVA-PF 10, pigmented), and CBIII (FPS 50 and UVA-PF 17, pigmented). The cream base CBII presented 15.86% of total chemical filters, which contain the following UV filters: ethylhexyl methoxycinnamate, bis-ethylhexyloxyphenol methoxyphenyl triazine—Tinosorb^®^ S, diethylamino hydroxybenzoyl hexyl benzoate—Uvinul^®^ A Plus, ethylhexyl triazone—Uvinul^®^ T150, methylene bis-benzotriazolyl tetramethylbutylphenol, and titanium dioxide. The CBIII presented 13.52%, composed of bis-ethylhexyloxyphenol methoxyphenyl triazine—Tinosorb^®^ S, diethylamino hydroxybenzoyl hexyl benzoate—Uvinul^®^ A Plus, ethylhexyl triazone—Uvinul^®^ T150, methylene bis-benzotriazolyl tetramethylbutylphenol, homoselate, and titanium dioxide. Additionally, CBII and CBIII presented pigments based on iron oxide (yellow, red, and black), which contribute to blocking high-energy visible light (400–500 nm) [39]. The complete description of the cream base compositions is presented in Table S1—Supplementary Materials.

### 2.13. Statistical Analysis

The results shown for the formulation’s physicochemical properties assessment were obtained as mean ± SD, using three replicates as original data. This data set was statistically evaluated by ANOVA and Tukey tests, using 95% confidence, and considered significantly different when *p* < 0.05. The data obtained for the skin permeation was based on non-parametric parameters (median and MAD—median absolute deviation) and was evaluated using the Kruskal–Wallis test in the software GraphPad Prism 9.0. Furthermore, this test was performed using four replicates. The in vivo photoprotection data (SPF and UVA-PF) were evaluated using the Brown–Forsythe and Welch ANOVA test followed by Dunnett’s T3 multiple comparisons test. All data presented here for the DoE studies were generated by the software Design Expert 13. In all tests, the used confidence interval was 95%, where *p*-values < 0.05 were considered significant.

## 3. Results

### 3.1. Formulation and Process Optimization

The production of the SC-NLC was conducted using as lipidic sources bacuri butter (solid lipid) and raspberry seed oil (liquid lipid), summing 10% of the formulation weight; the study of bacuri butter and raspberry seed oil is shown in the patent application number WO2023137532A1. They were chosen as lipid excipients due to their activity as antioxidants and moisturizers, as well as the sunscreen effect of the raspberry seed oil (*Rubus idaeus* L.) [40,41,42,43]. The latter is a yellowish and cloudy liquid of low viscosity (app. 26 mPas/s); it is mainly composed of fatty acids (app. 85%)—oleic, linoleic, and α-linoleic acids—and also contains a high percentage of vitamin E and tocopherols, which provides antioxidant activity to this oil [41,44]. The former is a butter extracted from an Amazonian fruit named bacuri (*Platonia insignis*). It is a yellowish solid with a melting point ranging from 40 to 60 °C, composed mostly of saturated, e.g., palmitic acid, and monounsaturated fatty acids, e.g., oleic acid, with antioxidant and anti-inflammatory properties [45].

In addition to the lipid components, a mixture of Uvinul^®^ A Plus, Tinosorb^®^ S, and Uvinul^®^ T150 at a ratio of 3:2:1 was used to develop a formulation that could provide protection against a broad range of the UV spectrum (UVA and UVB radiation) and achieve enhanced SPF [46,47]. The chemical filters ratio was selected with the aid of a simulator (DSM Sunscreen Optimizer), providing a filter composition that would achieve a theoretical value of 10 for both SPF and UVA-PF and a UVA/UVB photoprotection ratio of 1, which is above the 1/3 recommended by COLIPA [28,47]. The organic UV filters were encapsulated in NLCs to promote several upgrades in the sunscreen formulation, such as improvement in the filters’ chemical stability and extending the photoprotection effect [1,4,25]. 

To promote the particle’s stabilization, the non-ionic surfactants Polysorbate 60 and Polyglyceryl-3-dioleate were used. Surfactants play an important role in NLC’s colloidal properties, such as size and stability [48,49]. Non-ionic surfactants act as steric stabilizers, promoting mild colloidal stability, and their use has been increasing due to their low impact on physiological structures [48]. Since the identity and proportion of surfactants can have a significant impact on NLC’s size [48], we performed a 2^2^ Face Centered Central Composite Design to investigate the effect of Polysorbate 60 and Polyglyceryl-3-dioleate individually on NLC’s size and determine a desirable experimental region where NLCs would range between 100 and 300 nm. On the one hand, the upper level of the CQA (diameter < 300 nm) was defined based on the increased interaction that larger particles have with light, which would result in a formulation with an unwanted opaque and white-colored appearance. On the other hand, the lower CQA level was determined to avoid skin permeation since NLCs larger than this limit have negligible skin permeation [1,4,25].

The linear regression analysis of SC-NLC size showed that Polysorbate 60 and Polyglyceryl-3-dioleate have a significant negative (from 1682 to 202 nm) and positive effect on SC-NLC diameter (from 196.8 to 218.8 nm), respectively. The interaction between the surfactants and the quadratic term of Polysorbate 60 was also significant and negative. The positive effect promoted by the Polysorbate 60 ruled the SC-NLC size, superseding the opposite Polyglyceryl-3-dioleate effect. The high HLB value presented by Polysorbate 60 (14.9) indicates that this surfactant has a great interaction with water, and increasing its amount on the NLCs’ surface also leads to the generation of a thicker hydration layer [50]. This water structure provides protection to each NLC, hindering the interaction between particles and, hence, preventing their undesirable growth [50,51]. Several studies in the literature demonstrate that the use of polysorbates, in special Polysorbate 60, originates smaller and more homogeneous particles [52,53,54].

**Figure 2:**
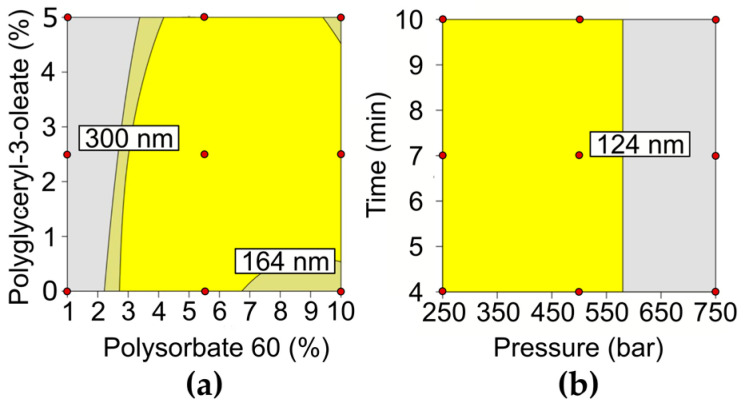
Design space (yellow region) of SC-NLC varying (**a**) Polysorbate 60:Polyglyceryl-3-dioleate ratio (**b**) and HPH parameters—pressure and time of processing.

The design space (DS) was obtained by the overlay of the contour plots of each statistically significant response; ICH defines it as “the multidimensional and interaction of input variables that have been demonstrated to provide assurance of quality of the product” [55]. Thus, the DS generated for SC-NLCs size shows the proportion of each surfactant that led to formulations (yellow region) that meet the CQA (see Figure 2a). The DS for formulations in the lower level of Polyglyceryl-3-dioleate (0%) ranges from 2.75% to 6.75% of Polysorbate 60. A broader range of the optimized region was reached when the Polyglyceryl-3-dioleate ratio was 2.5%, in which SC-NLC presenting less than 300 nm were produced in the range of 3 to 10% of Polysorbate 60. Additionally, the concentration of Polysorbate 60 must be increased to preserve the SC-NLC size under the CQA when the Polyglyceryl-3-dioleate amount increases from 0 to 5%. The larger particle diameter observed with higher amounts of Polyglyceryl-3-dioleate might be explained by the closer interaction of its lipophilic with the oily core once its HLB is between 4 and 5 [52]. In this way, the increase in Polyglyceryl-3-dioleate concentration could produce lipid core enlargement, as shown by Andreozzi et al. (2013) [56]. Although several formulations were part of the DS, the SC-NLC-PCs (central point) were elected as the optimized formulation. Regarding the proportion of surfactants, these samples had an average size of 205 nm (SD = 4.8 nm). This sample was applied as a standard for the factorial design of the SC-NLC production process, with the aim of translating the production process from ultrasonic to high-pressure homogenization (HPH) methods.

To optimize the process parameters, a 2^2^ CCF design was applied to define the conditions of homogenization time and pressure to be used on HPH (Table 2), using a CQA between 100 and 300 nm. The linear regression data showed that the linear and quadratic terms of homogenization pressure were statistically significant for the model, while the terms related to the homogenization time were disregarded from the model due to their low statistical significance. Thus, the quadratic model was generated to describe the SC-NLC’s size in this study and presented a determination coefficient (R^2^) of 0.88. As aforementioned, the homogenization pressure impacts significatively on SC-NLC’s diameter, and this effect is negative where the particle diameter goes from 134.7 to 199.9 nm when the pressure is dropped. The decrease in SC-NLC’s size responding to the rise in homogenization pressure is related to the shear force applied to the droplets. The shear force increases under high pressure and, hence, generates smaller particles [57]. 

For the DS definition, higher and lower limits were set for SC-NLC’s size, which were defined as 300 and 100 nm, respectively (Figure 2b). The minimum size level was set due to the enhanced skin permeation of NLCs whose sizes are under this limit [25]. Then, the obtained DS showed that formulations produced under homogenization pressure up to 575 bar yielded SC-NLC under 300 nm, as desired. Therefore, we were able to show that the HPH can successfully replace the ultrasound method for SC-NLC production. For further characterization and efficiency test purposes, the homogenization pressure and time chosen as optimal for the HPH process were 450 bar and 5 min, respectively. This experimental point was defined based on low equipment time requirement, and the pressure applied also avoids the extremes of the experimental region. As a result, the final optimized SC-NLC sample presented hydrodynamic diameter and PdI of 123 nm (SD = 0.50 nm) and 0.18 (SD = 0.01), respectively. 

### 3.2. SC-NLC Physicochemical Characterization

**Figure 3:**
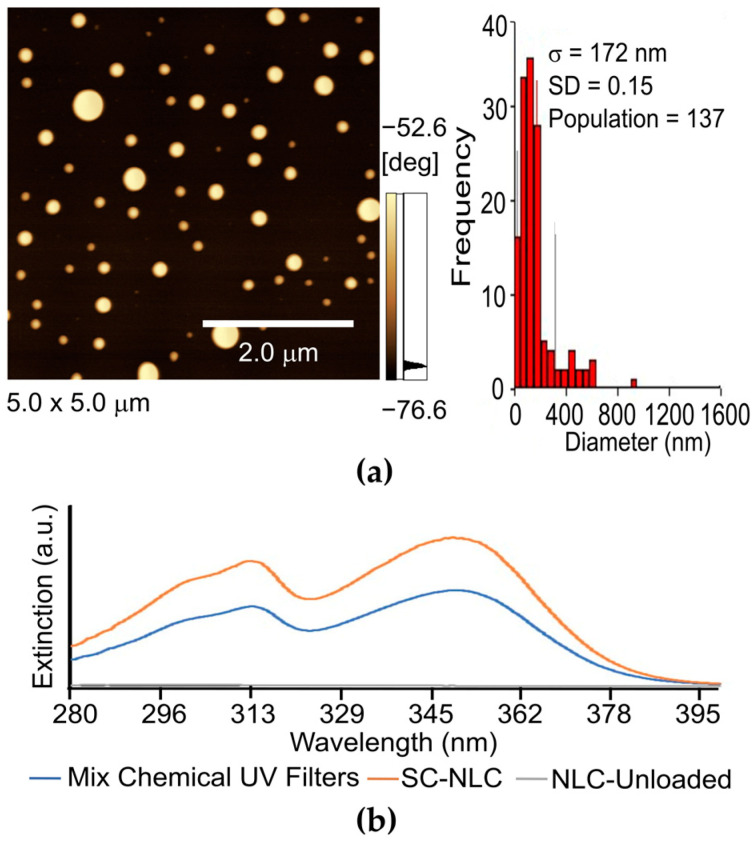
(**a**) AFM image, where the color scale shows that the brighter spots represent regions that are more exposed (in a higher position), and the darker fraction indicates the sample background. The scale shown in the image as reference is 2 µm. The histogram is resultant from a sum of several AFM images, showing the size frequency (non-normalized) of the optimized SC-NLC; (**b**) UV-Vis light extinction spectra of the mixtures of Uvinul^®^ A Plus, Tinosorb^®^ S, and Uvinul^®^ T150 free and encapsulated in the SC-NLC, also of the mixture of the SC-NLC unloaded (no filters).

Even though DLS informs the hydrodynamic diameter and parameters referent to colloidal behavior, it fails in defining the particle’s shape. For this purpose, the optimized SC-NLC was assessed by AFM, and as observed in Figure 3a, the produced particles presented a spherical shape and an average size of 172 nm (SD = 0.15). In addition to the particles’ morphology, the UV-Vis spectra of the chemical filters were also evaluated once their light absorption could be modified when closely interacting with SC-NLC’s lipidic components. Figure 3b shows that no shift or deformation of the chemical filters’ absorption bands was caused by their encapsulation. In fact, a noticeable increase in light absorption is presented by the sample of SC-NLC containing the chemical UV filters, which is due to the contribution of several mechanisms, including (a) the raspberry seed oil and bacuri butter UV-Vis absorption (Figure 3b) and (b) a constructive interaction between flavonoids contained in the lipid sources and the resonance structures present in the chemical filters molecules [58].

### 3.3. SC-NLC Physicochemical Stability

**Figure 4:**
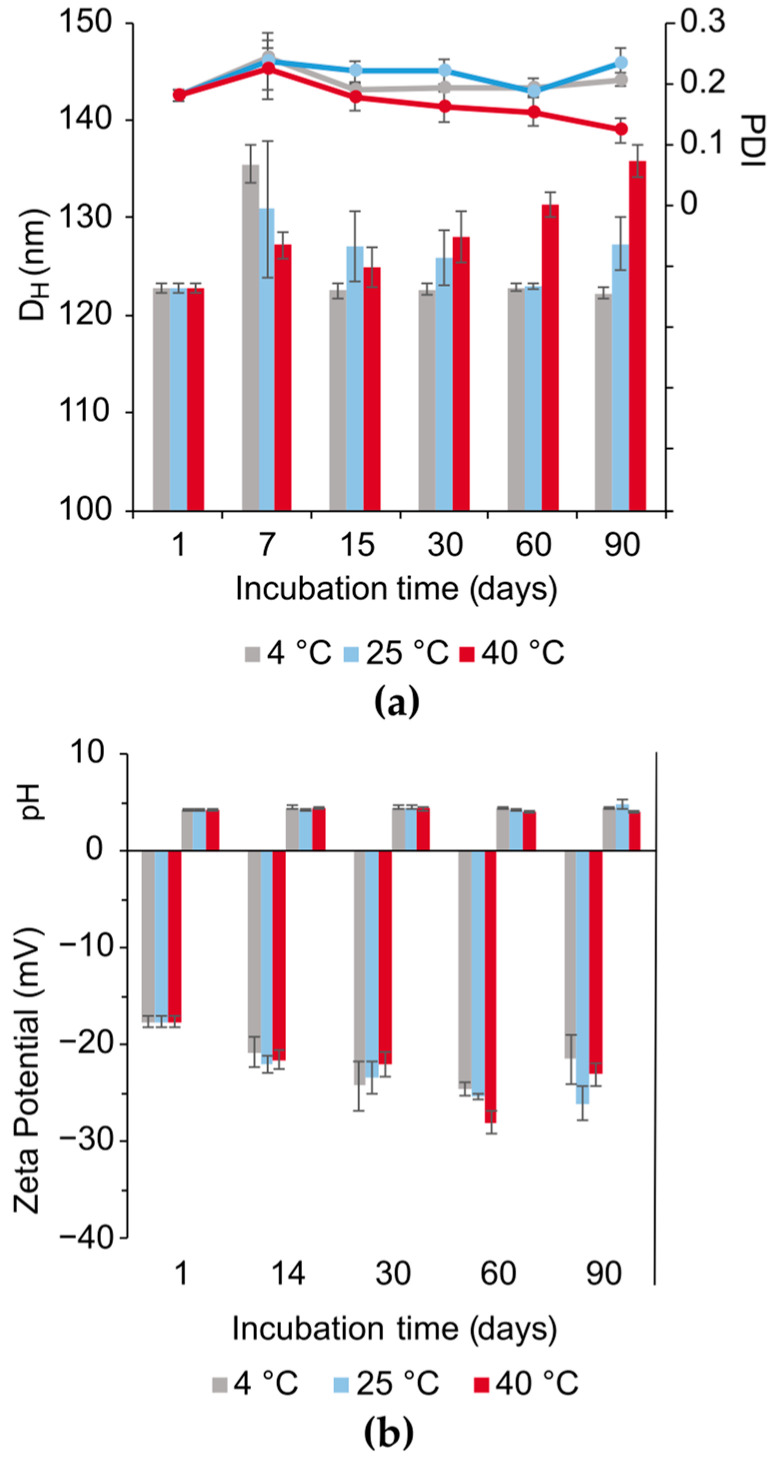
Evolution over time (in days) of SC-NLC’s (**a**) size (nm), PdI, (**b**) zeta potential (mV), and pH during three months under different incubation temperature conditions (4, 25, and 40 °C) and relative humidity of 75% (except for 4 °C). For the plot (**a**), the bars are referent to the particles’ hydrodynamic diameter (D_H_), and the lines express the PdI trend.

**Figure 5:**
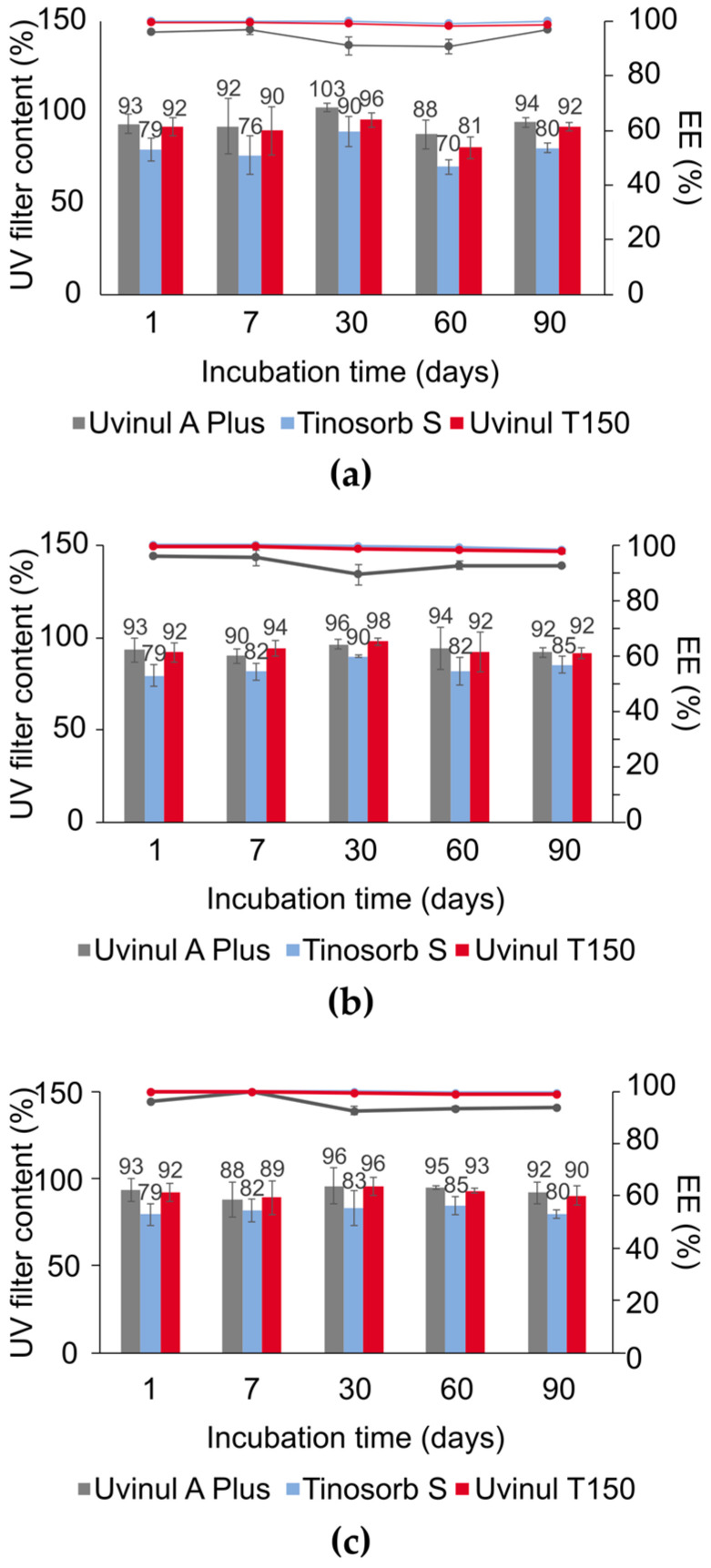
Percentage of UV filter total load and encapsulation efficiency for Uvinul^®^ A Plus, Tinosorb^®^ S, and Uvinul^®^ T150 at relative humidity (RH) of 75% and temperature of (**a**) 4 °C (refrigerator stability condition—RH not controlled), (**b**) 25 °C (long term stability condition), (**c**) 40 °C (accelerated stability condition). For all graphs the bars are referent to the percentage of filter content found in the SC-NLCs sample, and the lines express the percentual of filters encapsulated into the NLCs.

The SC-NLC’s physicochemical properties were evaluated over time in terms of hydrodynamic diameter, PdI, zeta potential, pH, total chemical UV filter load, and encapsulation efficiency, gauged during three months for the optimized SC-NLC submitted to the following conditions: (a) at 4 °C (relative humidity, RH, 70%); (b) at 25 °C (RH 75%); (c) at 40 °C (RH 75%). Figure 4 and Figure 5 compile the data collected during the stability assay, which was submitted to ANOVA and Tukey tests to identify significant differences (95% confidence) in the mean values of each group. The SC-NLC’s size and PdI present a slight variation throughout the 90 days under all conditions. For samples incubated at 40 °C, a gradual and significant increase in SC-NLC’s size was noticed at day 30 due to the droplet’s slight coalescence. Even though the size increment was shown to be statistically different after this time point, the total increase of cca 20 nm does not compromise the other physicochemical properties. Additionally, the PdI values for the samples at 40 °C dropped over each month; however, this is an apparent effect because the PdI is calculated based on Z_ave_ values (mean hydrodynamic diameter of a particle’s population—D_H_), where the increase in the later leads to a decrease in the former when no change is observed in the size distribution width. 

Zeta potential and pH values slightly varied during the 90 days, but these variations were not statistically significant (Figure 4b). The negative zeta potential found can be related to anionic species present in the lipidic sources, bacuri butter and raspberry seed oil, e.g., phenols, partial glycerides, and free fatty acids [49]. Since lipid degradation and severe colloidal destabilization would lead to changes in the formulation pH and zeta potential [55], these results indicate that the SC-NLC exhibited physicochemical stability. 

Figure 5 displays the results of UV filters’ total load and encapsulation efficiency. No significant loss of UV filter content was seen in any temperature condition during the first 90 days. For the evaluation of encapsulation efficiency (%EE), it was noticed that over 80% of each UV filter available in the formulation was entrapped into the SC-NLC’s structure throughout the entire experiment window and under all tested conditions. 

Furthermore, the total loading capacity (%LC) based on UV filter content and %EE of the three filters was determined following Equation (2). The SC-NLC showed a %LC of around 23% (cca 12%—Uvinul^®^ A Plus; cca 7%—Tinosorb^®^ S; cca 4%—Uvinul^®^ T150) in the three different incubation conditions for at least 90 days, presenting a loading capacity greater than previous studies using alternative lipid-based systems. For instance, Lúcio et al. (2021) found an %LC under 12% for liposomes, SLN, and NLCs encapsulating avobenzone, and Nikolìc et al. (2011) observed a loading capacity lower than 11% for NLCs encapsulating three chemical UV filters [20,33]. In conclusion, the stability tests indicate that the SC-NLC formulation presents good physicochemical stability.

### 3.4. In Vitro Skin Permeation Test 

**Figure 6:**
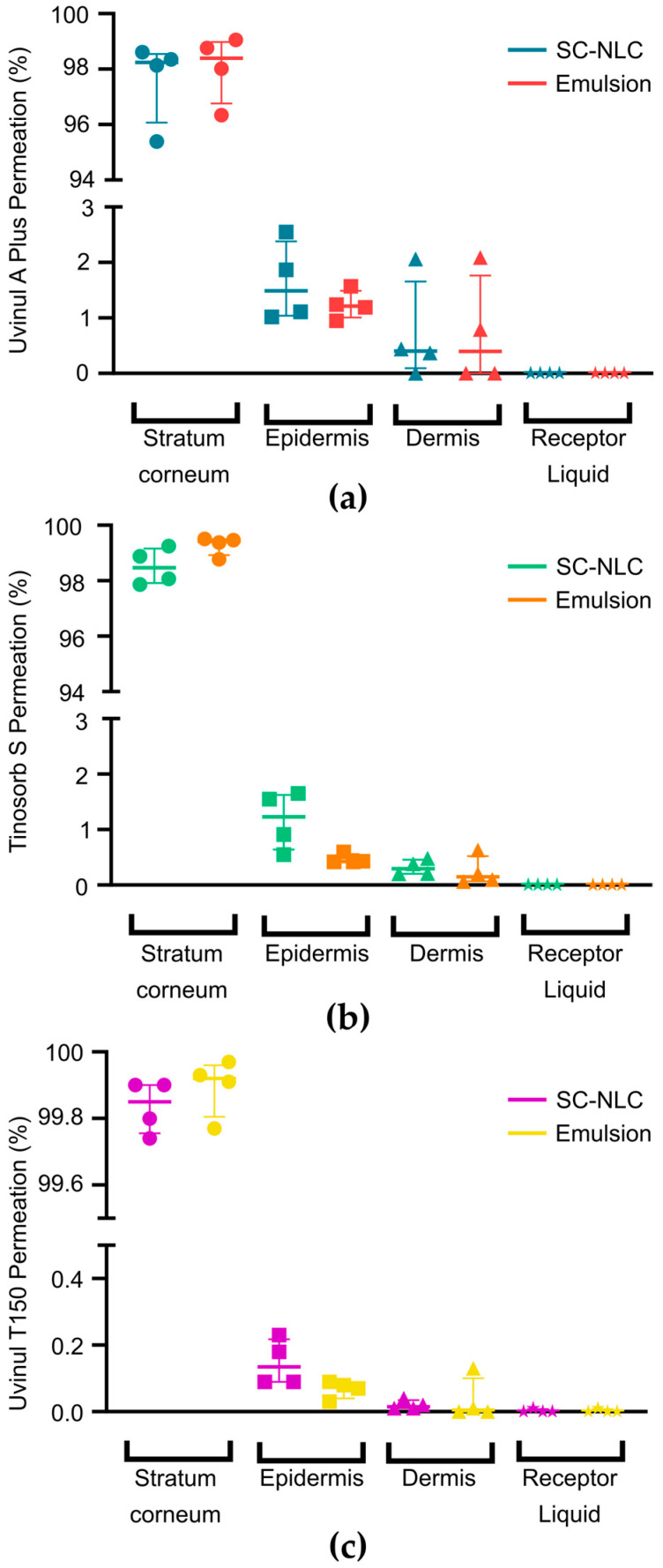
Permeation of (**a**) Uvinul^®^ A Plus, (**b**) Tinosorb^®^ S, and (**c**) Uvinul^®^ T150 into the stratum corneum, epidermis, dermis, and receptor liquid. This data set was statistically evaluated by the Kruskal–Wallis test and a significance threshold of less than 0.05.

Since the use of NLCs can prolong the skin residence time of sunscreen products by preventing transepidermal water loss [59], we sought to investigate whether this would impact the skin permeation of the filters in comparison to a pre-emulsion control (droplet size of approximately 5 µm). Figure 6 shows that for both samples, most of the filter content remained (>98%) in the skin surface and stratum corneum, in line with OECD 428 [36]. An equivalent permeation of all three filters was observed in the epidermis and dermis for both the SC-NLC and emulsion samples, and no filter was detected in the receptor liquid. In the epidermis, the concentration of Uvinul^®^ A Plus, Tinosorb^®^ S, and Uvinul^®^ T150 was, respectively, 1.49%, 1.23%, and 0.14% for the SC-NLC group, and 1.21%, 0.43%, and 0.08% for the emulsion sample. In the dermis, the amount of filters was 0.40% (Uvinul^®^ A Plus), 0.29% (Tinosorb^®^ S), and 0.01 (Uvinul^®^ T150) for the SC-NLC formulation, and 0.40% (Uvinul^®^ A Plus), 0.15% (Tinosorb^®^ S), and cca 0.00% (Uvinul^®^ T150) for the emulsion. The concentration values found in each skin explant are presented in Table 3. The Kruskal–Wallis statistical test confirmed that no significant difference was observed in the skin permeation of the three evaluated filters between the SC-NLC or emulsion formulations. Hence, the nanometric dimensions of the SC-NLC did not lead to increased skin absorption of the filters, making it safe for further topical use. Although previous studies have confirmed that only nanostructures smaller than 36 nm would be able to diffuse through the nanopores (5–7 nm in healthy individuals) of the stratum corneum structure [25,60,61], different mechanisms could allow the access of larger structures into deeper layers of the skin, such as the trans follicular route and extra skin hydration through occlusion. The latter could theoretically occur due to the nanoparticles’ presence, favoring skin penetration [25,62]. However, our findings do not corroborate this assumption, as also described by Puglia et al. (2014) [4]. The permeation observed for the SC-NLC was possibly hindered by the repulsion between the negative charges from the carrier surface (as indicated by the zeta potential data) and the stratum corneum lipid composition [59,61]. The stratum corneum acts as a highly efficient barrier that controls the penetration of molecules and microorganisms through the skin [3,25]. Additionally, the three chemical filters tested are highly lipophilic (logP > 5) and more likely to interact with the lipidic mass of the skin’s outer layer [63], and the molecular weight of Tinosorb^®^ S and Uvinul^®^ T150 is higher than 500 Da, hampering their skin penetration [64,65].

### 3.5. In Vitro Photoprotection Test

In vitro photoprotection screening was carried out to investigate the impact of the addition of SC-NLC on the FPS and UVA-PF values of a cream base (CB) sample. The following groups were investigated: CB (100% free chemical UV filters), SC-NLC (100% encapsulated filters), emulsion (100% encapsulated filters, obtained before the HPH process), and the CB containing 10, 20, and 30% (*w*/*w*) of SC-NLC or emulsion, respectively (Table 4). As the emulsion precedes the SC-NLC in the production process, they have the same composition and only differ in particle size matter (emulsion size > 3 µm, SC-NLC size < 150 nm). The CB was composed, respectively, of 1.5, 3.5, and 0.5% of free Uvinul^®^ A Plus, Tinosorb^®^ S, and Uvinul^®^ T150, and therefore, the combination of the CB with lipid additives (SC-NLC or emulsion) yielded final formulations with free and encapsulated chemical UV filters. 

The photoprotection data generated for the samples with increasing amounts of lipid additives (Table 4) showed that the SPF and UVA-PF increases provided by SC-NLC were greater than the ones of the emulsion controls. This result may be explained by the synergetic effect of light absorption, promoted by the chemical UV filters and the flavonoid molecules in the lipidic composition (as shown in Figure 3b), but also due to the scattering promoted by the SC-NLC’s core structure, composed mainly of bacuri butter [20,66]. Similarly, Nikolìc et al. (2011) observed that when NLCs containing carnauba wax were mixed in a base with free UV filters, a 45% increase in SPF was generated [20]. Although the emulsion presents micronized particles, the scattering effect produced by them is already found in the cream base composition. Hence, particles must be on the nanometer scale to achieve a significant scattering effect [67].

Subsequently, considering the SC-NLC’s superiority over the emulsions, the impact of their load on the CB’s SPF and UVA-PF was further gauged. Table 4 shows that samples with 20% (*w*/*w*) of SC-NLC resulted in the highest SPF (107.67 ± 17.15) and UVA-PF values (28.67 ± 2.05), which might be related to a fine balance between the reduction in chemical filters’ concentration and the inclusion of the SC-NLC. Surprisingly, the sample containing 20% of SC-NLC reached an SPF value comparable to the one observed for the 100% CB formulation (SPF of 111.33 ± 7.41, UVA-PF of 22.00 ± 1.41), and the response for UVA-PF was enhanced, despite the reduction in the total amount of chemical UV filters (Table 4). 

Table 4 also shows the values obtained for the UVA/UVB (UVA-PF/SPF) ratio, which must be at least one-third to meet the parameters set by COLIPA and FDA [24]. This guidance is based on the importance of guaranteeing skin protection against UVA radiation, which was neglected until the 2010s [28]. Since the resultant UVA/UVB ratios were nearly 0.3 for the formulations with 20% of SC-NLC, we proceeded with the in vivo photoprotection tests to confirm the photoprotective effects of the lead formulations and their UVA/UVB ratios in a more translational setting.

### 3.6. In Vivo Photoprotection Evaluation

We conducted an in vivo photoprotection evaluation of the lead formulations to confirm their enhanced photoprotection properties. For this study, two additional cream bases containing free chemical filters were used, labeled CBII and CBIII, to further explore the versatility of the NLCs in cream bases with different FPS and UVA-PF values, see Table 5. CBII and CBIII present a similar composition to CBI, but they are pigmented, due to containing iron and titanium oxides. Aiming to replicate the dilution caused by the addition of the SC-NLC or emulsion (20%, *w*/*w*) to the CBs, samples of 80% of CBs (I, II, or III) were obtained by adding 20% of purified water. The fluid flow and viscosity behavior of the samples CB-80% and containing SC-NLC were assessed aiming to guarantee that the addition of the lipid-base formulation does not interfere with the rheological properties of the CBs used, see Figure S1—Supplementary Materials. As expected, the flow and viscosity of the CBs were not impacted by the addition of the SC-NLC due to its high aqueous composition (>70%) and consequent low viscosity and Newtonian behavior, see Figure S2—Supplementary Materials. Hence, all samples presented a non-Newtonian and pseudoplastic behavior, which is indicated by the viscosity dropping and shear stress rising when the shear rate is increased (Figure S1) [68,69]. A similar result was reported by Souto and Müller (2006) in their study about the rheological behavior of SLNs in commercial cream bases [70].

**Figure 7:**
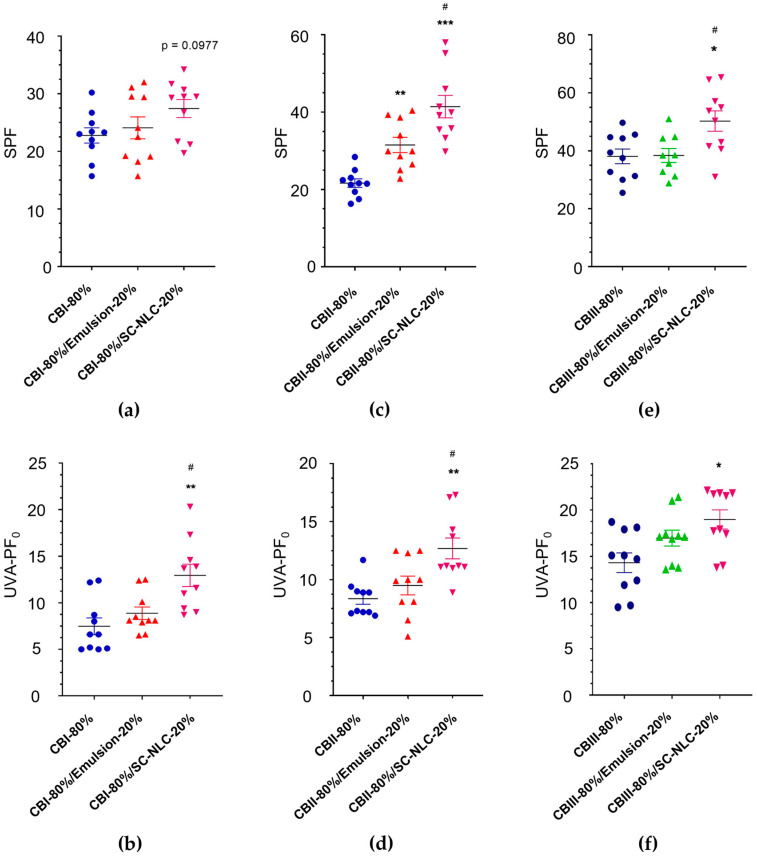
Photoprotection responses obtained in vivo tests assessing (**a**) SPF and (**b**) UVA-PF_0_ of CBI-80%, CBI-80%/Emulsion-20%, and CBI-80%/SC-NLC-20%; (**c**) SPF and (**d**) UVA-PF_0_ of CBII-80%, CBII-80%/Emulsion-20% and CBII-80%/SC-NLC-20%; and (**e**) SPF and (**f**) UVA-PF_0_ of CBIII-80%, CBIII-80%/Emulsion-20% and CBIII-80%/SC-NLC-20%. The results of SPF and UVA-PF_0_ are represented by their median, quartiles, and upper and bottom limits. The statistical difference was evaluated using the Brown–Forsythe and Welch ANOVA test followed by Dunnett’s T3 multiple comparisons test; it was considered significantly different when *p* < 0.05, and the symbols *, **, ***, and # indicate significant differences.

Figure 7 shows the data of the resultant SPF and UVA-PF for each CB-80%, CB-80%/Emulsion-20%, and CB-80%/SC-NLC-20%. For the analysis using the CBI and CBIII-based groups, a trend toward an SPF increase was seen for the SC-NLC-based formulation in comparison to their respective CB-80% formulation (*p* = 0.0977, for CBI-80%/SC-NLC-20%; *p* = 0.0358, for CBIII-80%/SC-NLC-20%), although they did not show difference when compared to their respective CB-100% (Figure 7a, Table 5). Thus, the addition of 20% of SC-NLC to CBI and CBIII overcame the reduction of 11.1 (CBI) and 15.3% (CBIII) on the amount of chemical UV filters, maintaining the values of SPF and UVA-PF like the ones found for CBI-100% and CBIII-100%. Surprisingly, for CBII, SC-NLC addition granted a significant increase of more than 30% in the SPF when compared to its respective 100% group (See Table 5, Figure 7c). The photoprotection enhancement observed after SC-NLC addition to a lipophilic cream base was also reported by Dario et al. (2018) [18]. As aforementioned, they found a 2-fold increase in the product’s SPF in the presence of 20% (*w*/*w*) of NLCs based on bocaiuva almond oil and cetyl palmitate (lipid sources) with the UV filters avobenzone and octocrylene [18]. Additionally, for these three cream bases, the SC-NLC effect promoted a greater impact on formulation SPF than the observed for the emulsion-containing samples, corroborating the phenomenon seen in the in vitro test. Similarly, the increment of UVA-PF values was statistically significant for CBI-80%/SC-NLC-20% (*p* = 0.0058—CBI-80%; *p* = 0.0296—CBI-80%/Emulsion-20%) and CBII-80%/SC-NLC-20% (*p* = 0.0022—CBII-80%; *p* = 0.0463—CBII-80%/Emulsion-20%) (Figure 7b,d). Furthermore, an increment on the UVA-PF_0_ of 27% (CBI-80%/SC-NLC-20%), 22% (CBII-80%/SC-NLC-20%), and 6% (CBIII-80%/SC-NLC-20%) was also detected when compared to their respective CB-100%, which contain a higher amount of chemical UV filters (See Table 5).

Finally, the SPF and UVA-PF were maintained or increased in the presence of SC-NLC, even though the formulations presented a lower content of chemical UV filters (Table 5). This response may have resulted from the combination of the nanoparticles with the antioxidant properties of the bacuri butter and raspberry seed oil [56]. The total percentage of chemical UV filters was reduced by 11.1% for CBI, 12.4% for CB-II, and 15.3% for CBIII when 20% of SC-NLC was added. Furthermore, the formulations CBI-80%/SC-NLC-20%, CBII-80%/SC-NLC-20%, and CBIII-80%/SC-NLC-20% meet the COLIPA recommendations and FDA criteria for UVA/UVB ratio (>0.33) [28]. 

As aforementioned in Section 2.12.2, the in vivo study was carried out with volunteers of different skin phototypes (SPT), using, respectively, phototypes II and III for SPF evaluation and phototypes III and IV for UVA-PF evaluation. These SPTs were selected based on their potential liability for each radiation type. Lighter skins are mostly impacted by UVB radiation, whereas darker skins have better protection against UVB but are more prone to develop hyperpigmentation upon overexposure to visible light and UVA [71]. The in vivo photoprotection results were also compared in a sub analysis dividing the data by each evaluated skin phototype. For both the SPF and UVA-PF analysis, the improved photoprotection promoted by the NLCs-containing formulations was overall consistent across the skin phototypes (Figure S3—Supplementary Materials).

## 4. Conclusions

The present study describes the development of novel sunscreen formulations utilizing SC-NLC (solid lipid nanoparticles) incorporating bacuri butter and raspberry seed oil (natural lipids) that efficiently encapsulate three chemical UV filters and promote enhanced photoprotection. This system showed high physicochemical stability for at least three months, preserving particle size, pH, zeta potential, and satisfactory %EE and UV filter content throughout the period of the test at temperatures of 4, 25, and 40 °C (RH of 75%). We also demonstrated that the presence of SC-NLC did not increase skin absorption of Uvinul^®^ A Plus, Tinosorb^®^ S, and Uvinul^®^ T150. Moreover, we found an optimum proportion of free and encapsulated filters that exhibited larger in vitro and in vivo SPF and UVA-PF than control emulsions across different cream bases, even with reduced chemical filter content. In summary, this study illustrates the integration of NLCs into sunscreen formulations to improve protection against UVB and UVA, simultaneously decreasing the overall content of organic filters and improving cosmeceutical properties.

## 5. Patents

Data shown in this manuscript have been used for the filing of the patent application number PCT/BR2022/050017.

## Figures and Tables

**Table 1:** Factor levels applied in the CCF designs for optimizing NLCs formulation (CCF-Formulation) and preparation process (CCF-Process).

Factor	−1	0	+1
CCF-Formulation			
Polysorbate 60 (%)	1.0	5.5	10.0
Polyglyceryl-3-dioleate (%)	0.0	2.5	5.0
CCF-Process			
Pressure (bar)	250	500	750
Time (min)	4	7	10

**Table 2:** 2^2^ Face Centered Central Composite Design for NLCs formulation varying the proportion of the surfactants Polysorbate 60 (x_1_) and Polyglyceryl-3-dioleate (x_2_), with proper randomized runs.

Sample	Run	x_1_	x_2_
SC-NLC-1	2	−1	−1
SC-NLC-2	1	+1	−1
SC-NLC-3	7	−1	+1
SC-NLC-4	3	+1	+1
SC-NLC-5	6	−1	0
SC-NLC-6	13	+1	0
SC-NLC-7	8	0	−1
SC-NLC-8	5	0	+1
SC-NLC-PC1	4	0	0
SC-NLC-PC2	12	0	0
SC-NLC-PC3	11	0	0
SC-NLC-PC4	9	0	0
SC-NLC-PC5	10	0	0

**Table 3:** Concentration of Uvinul^®^ A Plus, Tinosorb^®^ S, and Uvinul^®^ T150 detected in skin epidermis, dermis, and receptor liquid.

Filter	Epidermis (µg/cm²)		Dermis (µg/cm²)		Receptor Liquid (µg/cm²)	
	SC-NLC	Emulsion	SC-NLC	Emulsion	SC-NLC	Emulsion
Uvinul^®^ A Plus	15.86	12.95	4.31	4.24	0.00	0.00
Tinosorb^®^ S	8.51	3.32	2.03	1.16	0.00	0.00
Uvinul^®^ T150	0.50	0.29	0.05	0.02	0.00	0.00

**Table 4:** Lipid additive content (%, *w*/*w*), SPF, UVA-PF_0_, and UVA/UVB ratio values for the CB/SC-NLC, CB/Emulsion, pure CB, pure SC-NLC, and pure emulsion formulations obtained through the in vitro photoprotection test.

Sample	C_lipid aditive_ (%)	SPF	UVA-PF_0_	UVA/UVB
CB/SC-NLC	10	64.67 ± 8.50	24.67 ± 1.88	0.38
	20	107.67 ± 17.15	28.67 ± 2.05	0.27
	30	75.67 ± 10.21	18.33 ± 0.47	0.24
CB/emulsion	10	58.34 ± 7.36	18.33 ± 0.94	0.31
	20	57.00 ± 7.26	16.67 ± 1.70	0.29
	30	49.33 ± 6.94	18.00 ± 1.63	0.36
CB	0	111.33 ± 7.41	22.00 ± 1.41	0.20
SC-NLC	100	13.00 ± 1.41	10.67 ± 0.94	0.82
Emulsion	100	11.67 ± 1.24	8.33 ± 0.47	0.71

**Table 5:** Total chemical UV-filter content and in vitro and in vivo SPF and UVA-PF_0_ values for CBI, CBII, and CBIII at 80 and 100%, emulsion 20%, and SC-NLC 20% in the three tested cream bases.

Formulations	CB 100%			CB 80% ^1^			Emulsion 20% ^2^			SC-NLC 20% ^2^		
	I	II	III	I	II	III	I	II	III	I	II	III
Chemical filter content												
Total (%) ^3^	13.5	15.9	25.4	10.8	12.7	20.3	12.0	13.9	21.5	12.0	13.9	21.5
Filter content reduction (%) ^4^	-	-		-	-		11.1	12.4	15.3	11.1	12.4	15.3
SPF												
In vivo	30	30	50	23.2 ± 0.0	21.6 ± 2.8	38.1 ± 7.6	23.3 ± 5.6	29.9 ± 4.5	41.7 ± 12.0	29.4 ± 2.5	39.6 ± 5.2	50.3 ± 10.5
UVA-PF_0_												
In vivo	10.0	10.0	17.9	6.6 ± 1.6	8.1 ± 0.9	14.3 ± 3.4	8.1 ± 1.0	10.0 ± 2.1	16.9 ± 2.7	12.7 ± 1.5	12.2 ± 2.3	19.0 ± 3.3
UVA/UVB												
In vivo	0.30	0.30	0.36	0.28	0.38	0.38	0.35	0.33	0.41	0.43	0.31	0.38

^1^ 20% (*w*/*w*) of purified water added to the cream base; ^2^ 20% (*w*/*w*) of emulsion or SC-NLC in 80% of the cream base; ^3^ Chemical UV filter only; ^4^ Percentage calculated based on the CB 100%.

## Data Availability

The data presented in this study are available within the article.

## Supplementary Materials

The following supporting information can be downloaded at https://www.mdpi.com/article/10.3390/pharmaceutics16030427/s1, Table S1: List of components found in the cream base I, II, and III; Figure S1. Viscosity (Viscosity vs. Shear rate) and flow (Shear stress vs. Shear rate) curves of (a,b) CBI-80% and 20% of water (blank) or 20% of SC-NLC; (c,d) CBII-80% and 20% of water (blank) or 20% of SC-NLC; (e,f) CBIII-80% and 20% of water (blank) or 20% of SC-NLC; Figure S2. SC-NLC viscosity (a) and flow (b) curves, represented by viscosity vs. shear rate and shear stress vs. shear rate, respectively; Figure S3. SPF and UVA-PF response, respectively, based on the volunteer’s phototype applying cream base (a,b) I; (c,d) II, and (e,f) III, and their respective mixtures of 80% cream base + 20% of SC-NLC or 20% of emulsion.
