# Supplementary material for: NLC-Based Sunscreen Formulations with Optimized Proportion of Encapsulated and Free Filters Exhibit Enhanced UVA and UVB Photoprotection
Source: Pharmaceutics. 2024 Mar 20;16(3):427. doi: 10.3390/pharmaceutics16030427 (PMC10974804; doi:10.3390/pharmaceutics16030427)
Supplement: Supplementary file 1 [file pharmaceutics-16-00427-s001.zip › pharmaceutics-2872582-SI.pdf]

SUPPLEMENTARY MATERIAL

NLC-based sunscreen formulations with optimized proportion of encapsulated and free filters exhibit enhanced UVA and UVB photoprotection

Margarete M. de Araújo[1]†*, Andressa C. Schneid[1]†, Mariana S. Oliveira[2], Samuel V. Mussi[1], Miller N. de Freitas[3], Flávia C. Carvalho[4], Edson A. Bernes Junior[1], Renato Faro[5], Hatylas Azevedo[1]

1. Cream Bases Composition

The SC-NLC formulation was added into three cream bases (CBs), which were labeled as CBI, CBII and CBIII. On the Table S1 the composition of these CBs is decripted.

Table S1. List of components found in the cream base I, II and III.

Cream Base I	Cream Base II	Cream Base III
Water	Water	Water
Ethylhexyl methoxycinnamate	Aluminum Starch Octenylsuccinate	Ethylhexyl methoxycinnamate
C12-15 alkyl benzoate	Niacinamide	C12-15 alkyl benzoate
Cyclodextrin	Ethylhexyl salicylate	Aluminum starch octenysuccinate
Methyl methacrylate crosspolymer	C12-15 alkyl benzoate	Cyclopentasiloxane
Potassium cetyl phosphate	Homosalate	Glycerin
Glycerin	Pentylene glycil	Nylon-2
Stearyk Akcohol	Potassium cetyl phosphate	Potassium cetyl phosphate
Tribehenun PEG-20 esters	Silica	Tribhenin PEG-20 esters
Cetyl akcohol	Tapioca Starch	Cetearyl alcohol
Tocopheryl acetate	Tribehenin PEG-20 esters	Phenoxyethanol
VP/Eicosene Copolymer	Caprylyl glycol	Caprylyl glycol
Phenoxyethanol	Hydroxypropyl starch phosphate	Acrylates copolymer
Triethanilamin	Decyl glucose	Hydrated silica
Caprylyl glycol	Ethylhexylglycerin	Aluminum hydroxide

Methylpropional	Xanthan gum	Triethanolamine
BHT	Tocopheryl acetate	Acrylates/C10-30 alkyl acrylate crosspolymer
Benzyl Salicylate	VP/Eicosene copolymer	Decyl glucoside
Hexyl cinnamal	Sterаryl alcohol	Parfum
Citronellol	Talc	Alginic acid
Hydroxyethyl methylthiopropenamide	Methylpropanediol	Disodium EDTA
Myristyl alcohol	Cetyl alcohol	Butylphenyl methylpropional
Lauryl alcohol	Disodium EDTA	Decarboxy carnosine HCl
Bis-ethylhexyloxyphenol methoxyphenyl triazine	Aluminum hydroxide	Propylene glycol
Diethylamino hydroxybenzoyl hexyl benzoate	Stearic acid	Butylene glycol
Ethylhexyl triazone	Sodium C14-16 oledin sulfonate	Xanthan gum
	Gossypium hirsutum extract	Benzyl salicylate
	Butylene glycol	PEG-12 dimethicone
	Disodium phosphate	Hexyl cinnamal
	Propylene glycol	Citronellol
	Phenoxyethanol	Iron oxide
	Arachidyl alcohol	Titanium oxide
	Hydroxyethyl methylthiopropenamide	Bis-ethylhexyloxyphenol methoxyphenyl triazine
	Myristyl alcohol	Diethylamino hydroxybenzoyl hexyl benzoate
	Lauryl alcohol	Ethylhexyl triazone
	Iron oxide	Methylene bis-benzotriazolyl tetramethylbutylphenol
	Titanium oxide	
	Ethylhexyl methoxycinnamate	
	Bis-ethylhexyloxyphenol methoxyphenyl triazine	
	Diethylamino hydroxybenzoyl hexyl benzoate	
	Ethylhexyl triazone	
	Methylene bis-benzotriazolyl tetramethylbutylphenol	

2. Rheology

The Figure SI1 shows the flow and vicosity curves of the mixtures composed by 80% of cream base (I, II and III) and 20% of water (blank) or 20% of SC-NLC. Also, Figure SI2 present the SC-NLC flow and viscosity curves.

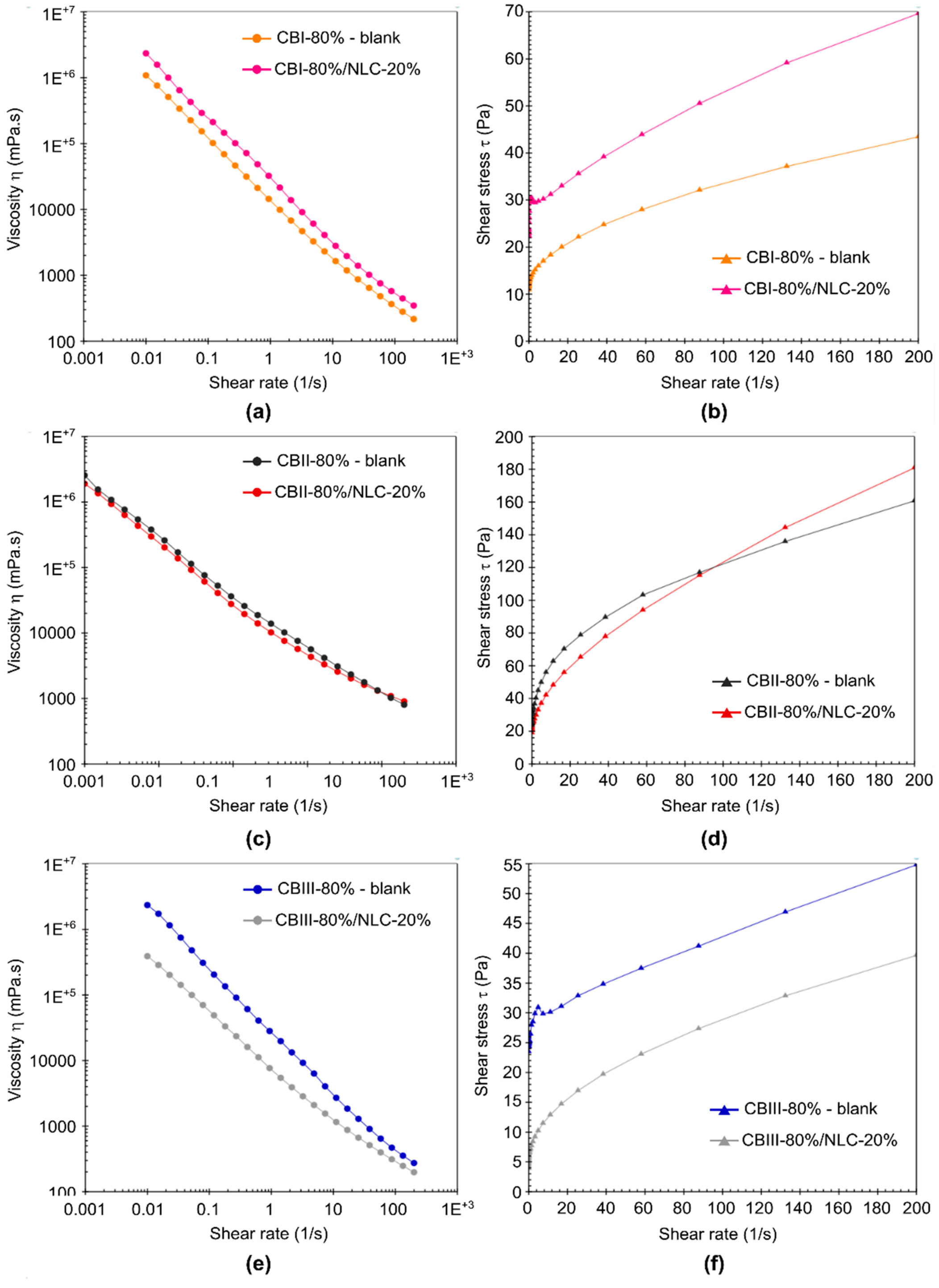

Figure S1. Viscosity (Viscosity vs Shear rate) and flow (Shear stress vs Shear rate) curves of (a - b) CBI-80% and 20% of water (blank) or 20 % of SC-NLC; (c - d) CBII-80% and 20% of water (blank) or 20 % of SC-NLC; (e - f) CBIII-80% and 20% of water (blank) or 20 % of SC-NLC.

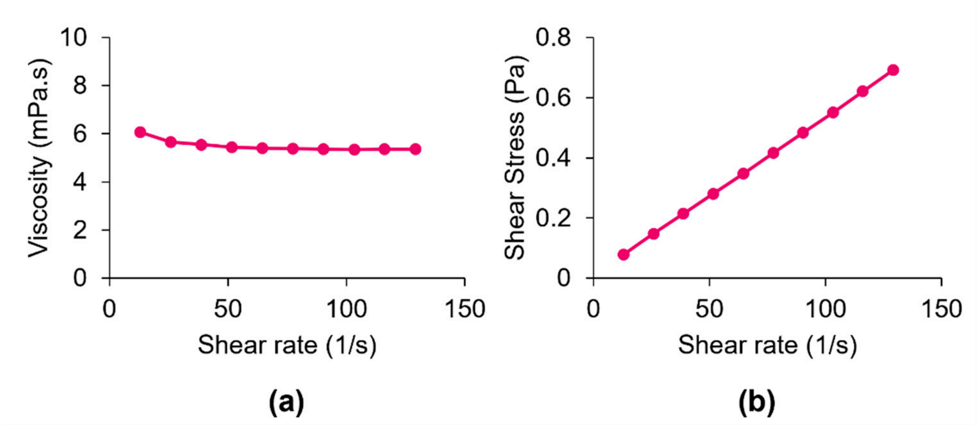

Figure S2. SC-NLC viscosity (a) and flow (b) curves, represented by viscosity vs shear rate and shear stress vs shear rate, respectively.

3. *In vivo* photoprotection

The FPS and UVA-PF data obtained for the three cream bases (I, II and III) containing 20% of SC-NLC or 20% of emulsion were evaluated based on the volunteers' skin phototype. This assay was performed aiming to better understand its impact on the products' photoprotection response. The results of this sub-analysis are shown in Figure S3.

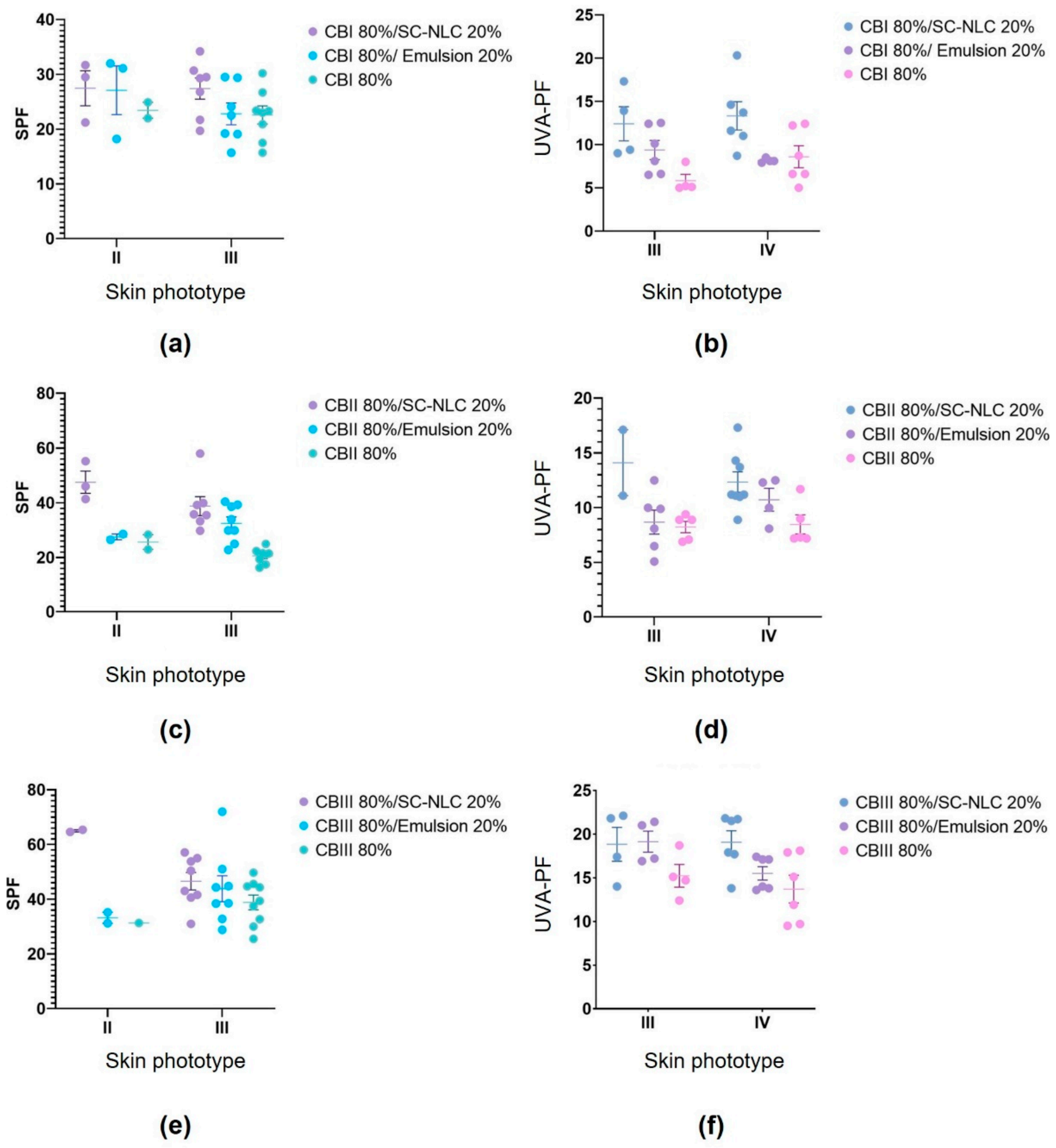

Figure S3. SPF and UVA-PF response, respectively, based on the volunteers phototype applying cream base (a-b) I; (c-d) II and (e-f) III, and their respectives mixtures of 80% cream base + 20% of SC-NLC or 20% of emulsion.
